# A diffusion tensor-based method facilitating volumetric assessment of fiber orientations in skeletal muscle

**DOI:** 10.1371/journal.pone.0261777

**Published:** 2022-01-27

**Authors:** Laura Secondulfo, Melissa T. Hooijmans, Joep J. Suskens, Valentina Mazzoli, Mario Maas, Johannes L. Tol, Aart J. Nederveen, Gustav J. Strijkers

**Affiliations:** 1 Department of Biomedical Engineering and Physics, Amsterdam UMC, University of Amsterdam, Amsterdam, The Netherlands; 2 Department of Radiology and Nuclear Medicine, Amsterdam UMC, University of Amsterdam, Amsterdam, The Netherlands; 3 Department of Orthopedic Surgery, Amsterdam UMC, University of Amsterdam, Amsterdam, The Netherlands; 4 Department of Radiology, Stanford University, Stanford, California, United States of America; Henry Ford Health System, UNITED STATES

## Abstract

**Background:**

The purpose of this study was to develop a DTI-based method to quantitatively assess fiber angles and changes therein in leg muscles in order to facilitate longitudinal studies on muscle fiber architectural adaptations in healthy subjects.

**Methods:**

The upper legs of five volunteers were scanned twice on the same day. The right lower legs of five volunteers were scanned twice with the ankle in three positions, *i*.*e*. -15° dorsiflexion, 0° neutral position, and 30° plantarflexion. The MRI protocols consisted of a noise scan, a 3-point mDixon scan and a DTI scan. Fiber-angle color maps were generated for four muscles in the upper legs and two muscles in the lower leg. Voxel-wise fiber angles (θ) were calculated from the angle between the principal eigenvector of the diffusion tensor and a reference line defined between the origo and insertion points of each muscle. Bland-Altman analysis, intraclass correlation coefficient (ICC), coefficient of variation (CV%), minimal detectable change (MDC), standard error (SE) and Friedman test were used for assessing the feasibility of this method and in order to have an indication of the repeatability and the sensitivity.

**Results:**

Bland-Altman analysis showed good repeatability (CV%<10 and 0.7≤ICC≤0.9) with exception of the Tibialis Anterior (TA) muscle in dorsiflexion position(CV%: 12.2) and the Semitendinosus (ST) muscle (left leg) (CV%: 11.4). The best repeatability metrics were found for the SOL muscle in neutral position (CV%: 2.6). Changes in average θ in TA and SOL with ankle positions were observed in accordance with expected agonist and antagonist functions of both muscles. For example, for the anterior left compartment the change in fiber angle Δθ with respect to the neutral position Δθ = -1.6° ± 0.8° and 2.2° ± 2.8° (p = 0.008), for dorsiflexion and plantarflexion, respectively.

**Conclusion:**

Our method facilitates fast inspection and quantification of muscle fiber angles in the lower and upper leg muscles in rest and detection of changes in lower-leg muscle fiber angles with varying ankle angles.

## Introduction

Muscle fiber architecture is a dominant determinant of muscle functioning in terms of tension, exertable force, response to physical exercise, as well as vulnerability to muscle injury and disease [1]. Biomechanical metrics for muscle force prediction include muscle contractile volume, fiber length and fiber angles (pennation angles). The study of changes in muscle architecture following intervention, training and response to therapy allows for a better understanding of the relationship between muscle architectural adaptations and function [2, 3].

In clinical practice, the most commonly used definition of pennation angle is the two dimensional (2D) pennation angle between the muscle fascicles and the aponeurosis or tendon [4] in a specific location of the muscle. Another used definition of pennation angle in 2D is the angle between the muscle fascicles and the line of action of the tendon [4]. This definition is useful but it lacks tridimensional information of the full muscle volume. A quantitative volumetric assessment is needed when there is no a priori knowledge concerning the location of expected changes in muscle architecture due to training or response to treatment. Less common in clinical practice but very useful in biomechanics for the estimation of muscle force are the definitions of pennation angle in three-dimensional volume, in which the pennation angle is defined between the muscle fascicles and the line of action of the muscle [5] which in turn can be subject to different definitions.

Traditional 2D ultrasound presents technical limitations which impede a straightforward volumetric assessment of the muscle architecture. In the last decade newer techniques have been introduced in order to obtain 3D muscle images of muscle fibers such as three-dimensional ultrasonography (3DUS) [5–7] and diffusion tensor imaging (DTI) in magnetic resonance imaging (MRI) [8].

Magnetic resonance imaging (MRI) and particularly diffusion tensor imaging (DTI) allows for a non-invasive quantification of muscle macro- and microstructural parameters [8]. Especially, in sport medicine, DTI has shown promise as a relevant tool to understand the muscle recovery process that follows physical activity and after injury [9]. Moreover, the inherent 3D nature of MRI facilitates the assessment of muscle fiber architecture in a volumetric way, for the majority of the body’s muscles.

DTI has been used previously for the quantification of muscle fiber angles according to different definitions. These studies showed congruity between the direction of the first eigenvector of the diffusion tensor ε_1_ and histology-defined fiber angles in skeletal [10–13] and cardiac muscle [14, 15]. Over the past decade various studies focused on estimating pennation angles in individual muscles based on DTI-based fiber tractography, different line of action definitions and manual aponeurosis delineation [16–18]. Fiber tractography is a non-invasive 3D modeling technique used to investigate the muscle microstructure, local collagen fiber alignment, and the 3D collagen network. It is obtained from DTI by combining the main diffusion direction ε_1_ and the fractional anisotropy FA of adjacent voxels in order to obtain tracts representing the muscle fibers and based on both deterministic and probabilistic algorithms generally relying on assumptions concerning the muscle physiology that translates in stopping criteria like the step size, the turning angle, the FA range, tract density and the maximum tract length. However, accurate and reproducible aponeurosis delineation on MR images is difficult [19] and fiber tractography outcomes are promising but subject to variability due to different algorithms and the stopping criteria. Furthermore, these stopping criteria and tracking settings often require optimization for individual subjects, muscles [16, 20, 21] and locations within the muscle which is a time-consuming process. This hampers reproducibility of the pennation angle measurements, particularly needed in longitudinal studies. Consequently, there is a need for a simple, semi-automated and robust method to quantify pennation angle in skeletal muscle. Ideally, such a method should be independent on the positioning of the subject in the MRI scanner, easily applicable, objective and based on non-deformable anatomical structures. Such a method will specifically facilitate longitudinal studies on muscle fiber architectural adaptations due to training and response to treatment.

The purpose of this study was to develop and evaluate the feasibility of a DTI-based method to quantitatively assess fiber angles and changes therein in whole leg muscle volumes in a straightforward manner, without the need to perform fiber tractography or tendon delineation, in order to facilitate longitudinal studies on muscle fiber architectural adaptations due to training or response to treatment.

## Methods

### Study design

We evaluated the repeatability and sensitivity of the method in the upper (experiment 1) and lower leg (experiment 2) muscles. The study was approved by the local institutional research board IRB and the medical research committee of the Amsterdam UMC and written informed consent was provided by all subjects prior to the study.

### Experiment 1 (Upper leg)

The upper legs of five (N = 5) healthy volunteers (3 male, 2 female, age range 20 to 40) were scanned twice on the same day with a 3T Philips Ingenia MRI scanner (Philips Healthcare, Best, the Netherlands), using a 16-channel anterior coil and the 10-channel table posterior coil. Subjects were positioned in feet-first supine position. The data were acquired in three transverse stacks with 30 mm overlap, covering 498 mm proximal to distal with a field of view (FOV) of 480 x 276 mm^2^. The MRI protocol consisted of a noise map to calculate signal-to-noise ratios (SNR) (SE-EPI without diffusion weighting, *T*_R_ = 4630 ms, *T*_E_ = 53 ms, matrix = 160 × 160, voxel size = 3 × 3 × 6 mm^3^, number of slices = 31, SENSE = 1.5), a 3-point mDixon scans as anatomical reference (sequence = FFE, TR = 8.0 ms, TE1/ΔTE = 1.33/1.1 ms, voxel size = 1.5 x 1.5 x 3.0 mm^3^) and a DTI scan for diffusion parameter estimation (sequence = SE-EPI, TR = 4630 ms, TE = 53 ms, voxel-size = 3 x 3 x 6 mm^3^, SENSE acceleration factor = 1.5, number of gradient directions = 48, diffusion b-value = 450 s/mm^2^). Slice-selection gradient reversal (SSGR) and spectrally adiabatic inversion recovery (SPAIR) were used for fat suppression [22]. The scan time per each stack was 7 min for the DTI scan, 2 min for the Dixon scan and 30 sec for the Noise scan. The participants were taken out of the MRI scanner in between the two sessions for about 15 minutes to rest.

### Experiment 2 (Lower leg)

**Fig 1:**
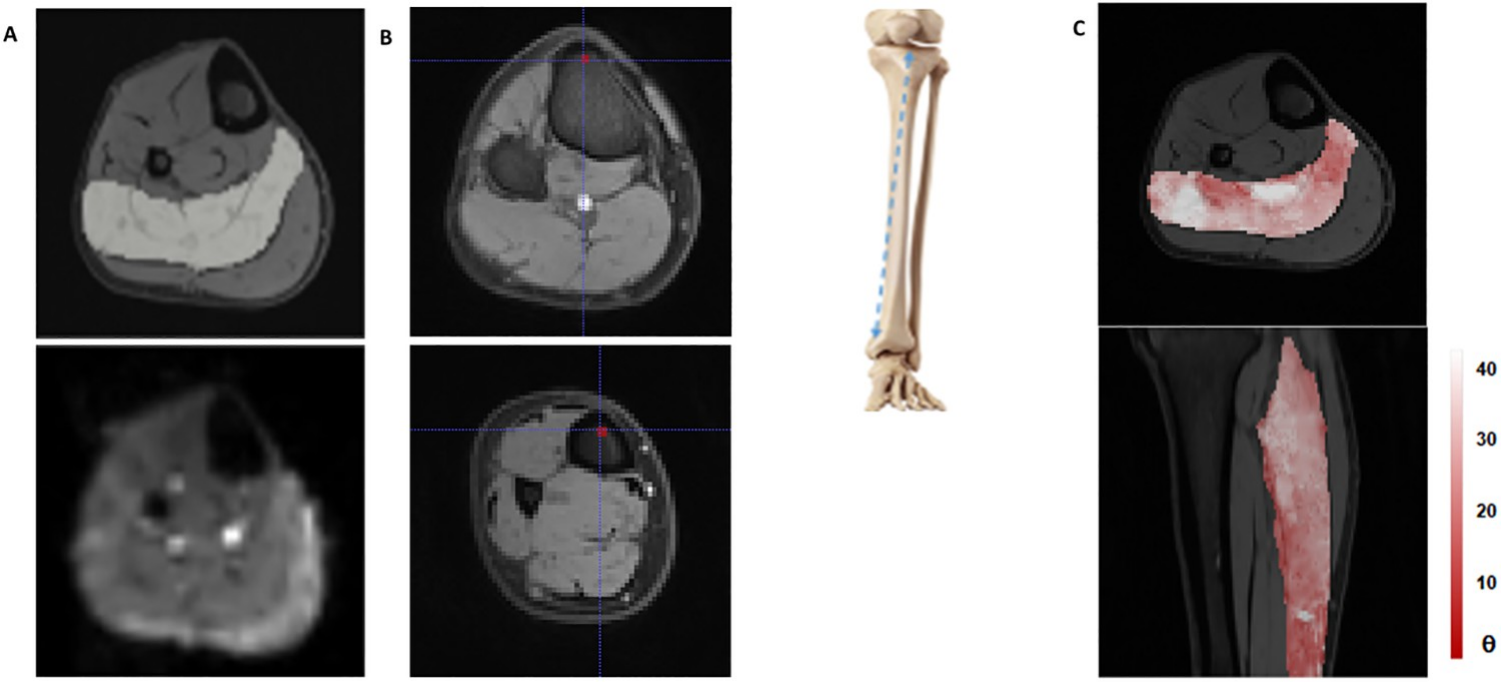
Graphical representation of steps to create 3D angle (θ) color maps. **(A)** An mDixon scan (top) were used as anatomical reference to segment the muscles. Example diffusion-weighted image (bottom) with b-value = 450 s/mm^2^. DTI was used for voxel-wise calculation of the principal diffusion direction, corresponding to the muscle fiber direction. **(B)** Reference points and schematical drawing of the reference line. The fiber angle was defined as the angle of the principal diffusion direction with the reference line, which in this example was defined by manually drawing points to anatomical landmarks (indicated by the blue crosshair and red points) on the tibia bone on the mDixon images. **(C)** Axial and longitudinal cross sections of the lower leg with the fiber angles of the SOL muscle as a color-coded overlay.

The right lower legs of five (N = 5) male healthy volunteers (age range: 21–43) were scanned with a 3T Philips Achieva MRI scanner (Philips Healthcare, Best, the Netherlands) using a 16-channel torso coil. A custom-built device was used to place the ankle in three different positions, *i*.*e*. -15° dorsiflexion, 0° neutral, and 30° plantarflexion, to induce passive lengthening and shortening of the muscles in the lower leg. MRI measurements of the lower leg for the three ankle positions were performed in one examination. The MRI protocol consisted of a 3-point mDixon scan (sequence = FFE, TR = 7.7 ms, TE1/ΔTE = 2.1/1.7 ms, voxel-size = 1.0 x 1.0 x 2.5 mm^3^, FOV = 192x156 mm^2^), the DTI scan (sequence = SE-EPI, TR = 11191 ms, TE = 51.63 ms, voxel-size = 3 x 3 x 5 mm^3^, SENSE acceleration factor = 1.5, number of gradient directions = 12, diffusion b-value = 400 s/mm^2^, FOV = 192x156 mm^2^) and a noise scan obtained by repeating the DT-MRI scan with a single volume and setting the power of the RF to zero. Slice-selection gradient reversal (SSGR) and spectrally adiabatic inversion recovery (SPAIR) were used for fat suppression [22]. The scan time for each ankle position was 11 min. Example anatomical reference and diffusion images for the lower leg are shown in Fig 1. Each volunteer was examined on the same day in two separate scanning sessions, with at least 30 min in between. The dataset was also used to assess fiber length by Mazzoli *et al*. [23].

### DTI post-processing

The DTI data were analyzed with QMRITools for Wolfram Mathematica (Wolfram Research, Inc., Mathematica, Version 12, Champaign, IL) [24]. Data processing consisted of 1) noise suppression, with Principal Component Analysis algorithm (PCA), 2) affine registration of the diffusion-weighted images to the non-weighted diffusion images to correct for eddy current-induced image distortions 3) b-spline registration to the mDixon water anatomical images to correct for susceptibility induced deformations [25]. Directional diffusion data were fitted to a tensor model with an iterative weighted linear least square algorithm (iWLLS) [26]. The principal eigenvector ε_1_ of the diffusion tensor in each voxel, corresponding to the muscle fiber angles, was calculated by an eigen decomposition. The SNR was calculated as the mean of the signal in a muscle ROI divided by the standard deviation of the noise (σ) as calculated from the noise scan. Datasets with SNR below 25 were excluded from further analysis [26].

### Segmentation

In the lower leg, to facilitate segmentations, the mDixon out‐of‐phase images were re-sampled to a resolution of 1 x 1 x 1 mm^3^. Manual delineation of the muscles was done in the resulting 25 slices using ITK-SNAP [27] on the images obtained with the ankle in neutral position. The segmentations were then transferred to all six datasets (three positions x two measurements) by registering the down‐sampled mDixon images to full resolution out‐of‐phase mDixon images using rigid registration followed by non‐linear b‐spline registration, as previously described by Mazzoli *et al*. [23].

For the upper leg, we segmented the biceps femoris long head (BFL) and the semitendinosus (ST) muscles. Additionally, the maps of the upper legs were divided in 5 proximal to distal sub-regions of equal length, *i*.*e*., 0–20%, 20–40%, 40–60%, 60–80%, and 80–100% of the muscle length by using Matlab R2016b (MathWorks, Natick, MA, USA). For the lower leg, the Soleus (SOL) and the Tibialis Anterior (TA) muscles were selected, because these are antagonist muscles and we expect differences in fiber angles with different ankle positions. Fig 1 illustrates the segmentation of the SOL with the ankle in neutral position. For a more detailed regional analysis of muscle fiber angles in a multi-pennate muscle, the SOL muscle was further segmented into four compartments, *i*.*e*., two anterior bi-pennate compartments and two posterior uni-pennate compartments, following anatomical landmarks such as tendon sheets and muscle borders. The segmentation was manually executed by LS (four years of experience) in the upper legs muscles and in the compartments of the lower leg. The full muscle volumes of the lower leg muscles were manually drawn by VM (seven years of experience). The manual delineation took about three hours per muscle.

### Fiber angle color maps

To calculate fiber angles and generate color maps of the fiber angles (θ) in the full muscle volume, the following strategy was applied. First, a reference line was defined in ITK-SNAP based on two anatomical landmarks identified in the out-of-phase images by a trained person according to a muscle specific definition, as schematically illustrated in Fig 1. For the upper leg (experiment 1), two reference lines were defined between the origo and insertion points of the BFL and the ST, respectively (Fig 1A), according to the definition of line of action of a muscle by Delp *et al*. [28] For the lower leg (experiment 2), a single reference line was defined for both the SOL and the TA muscles by a point on the tibia plate and the most distal and posterior point of the diaphysis (Fig 1B). The intra-observer variability of the fiber angle maps deriving from the placement of the reference line was evaluated.

The voxel-wise fiber angles (θ) were subsequently calculated in MATLAB R2016b (The MathWorks Inc., Natick, MA, USA) from the direction of the principal eigenvector of the diffusion tensor ε_1_ and the vector describing the direction of the reference line A using $Ɵ=acos\;(\frac{AƐ1}{\left|A||Ɛ1\right|})$, with the constraint of Ɵ<90° in order to ensure unique angles.

### Statistical analysis

Bland-Altman analysis, the Coefficient of Variation (CV%) and the Intraclass Correlation Coefficient (ICC) were used to represent and to assess the repeatability of the fiber angles maps quantified with our analysis method and expressed as mean value for the full muscle volumes of twenty and thirty individual upper and lower leg muscles respectively. The CV% is defined as the standard deviation divided by the mean [29]. An intra-subject CV% < 10 was considered an index of good repeatability. The ICC is defined as the variance of intra-subjects measurements divided by the total variance of inter-subjects and intra-subjects measurements and in this study for test-retest reliability it was calculated with a two-way random effects, with measures of absolute agreement [30]. Values of ICC between 0.6 and 0.75 were considered moderately good, 0.75≤ICC≤0.9 was considered very good, ICC>0.9 was judged very good, and ICC = 0.9 was rated excellent. The Bias is calculated as the mean of the errors between the two measurements. In this context the bias does not represent the trueness of the method in respect to a gold-standard [31], but it is intended as a measure of the repeatability of the method. The coefficient of repeatability CR can be calculated as 1.96 (or 2) times the standard deviation of the differences between the two measurements, it is a precision measure which represents the value below which the absolute difference between two repeated test results may be expected to lie with a probability of 95%. Additionally also the Standard Error of the estimate (SE) and the Minimal Detectable Difference(MDD) were calculated in order to compare with other studies. The SE is calculated by taking the standard deviation and dividing it by the square root of the sample size [32], it indicates how large the prediction errors of the estimates is for the dataset [33]. MDD is measured as the standard error of measurement multiplied by 1.96 and √2 [30]. The MDD indicates the smallest change that can be detected statistically.

Fiber angle maps and distribution plots are used to evaluate the ability of the method to assess differences in fiber angles between the three ankle positions in the full volumes of the SOL and the TA muscles and in the four muscle compartments of the SOL muscle. Furthermore, Friedman tests were used to assess differences in fiber angles between the three ankle positions in the four muscle compartments of the SOL muscle. Post-hoc analysis with Wilcoxon signed ranks test was used to determine which of the compartments differed in fiber angles. All statistical analysis were performed in SPSS (IBM SPSS Statistics for Windows, Version 26 Armonk, NY: IBM Corp) and the significance level was corrected for multiple comparisons and set at p = 0.017.

## Results

All scans were successfully completed and all DTI datasets had signal to noise ratio SNR > 25, to allow for accurate tensor calculations [26]. The average SNR in the muscles in the non-diffusion-weighted images in experiment 1 (upper legs) was above 25 (range 25–70), whereas for experiment 2 (lower legs) it was above 30 (range 30–70). The fiber angle color maps were calculated and Fig 1C shows representative cross-sections of the lower leg with overlays of the SOL muscle, color-coded according to the fiber angle (θ) to illustrate the 3D nature of our approach.

The intra-observer variability for placing the reference line in the BF, ST and lower leg were 1.0±0.4 voxels and 0.4±0.6 voxels respectively.

**Fig 2:**
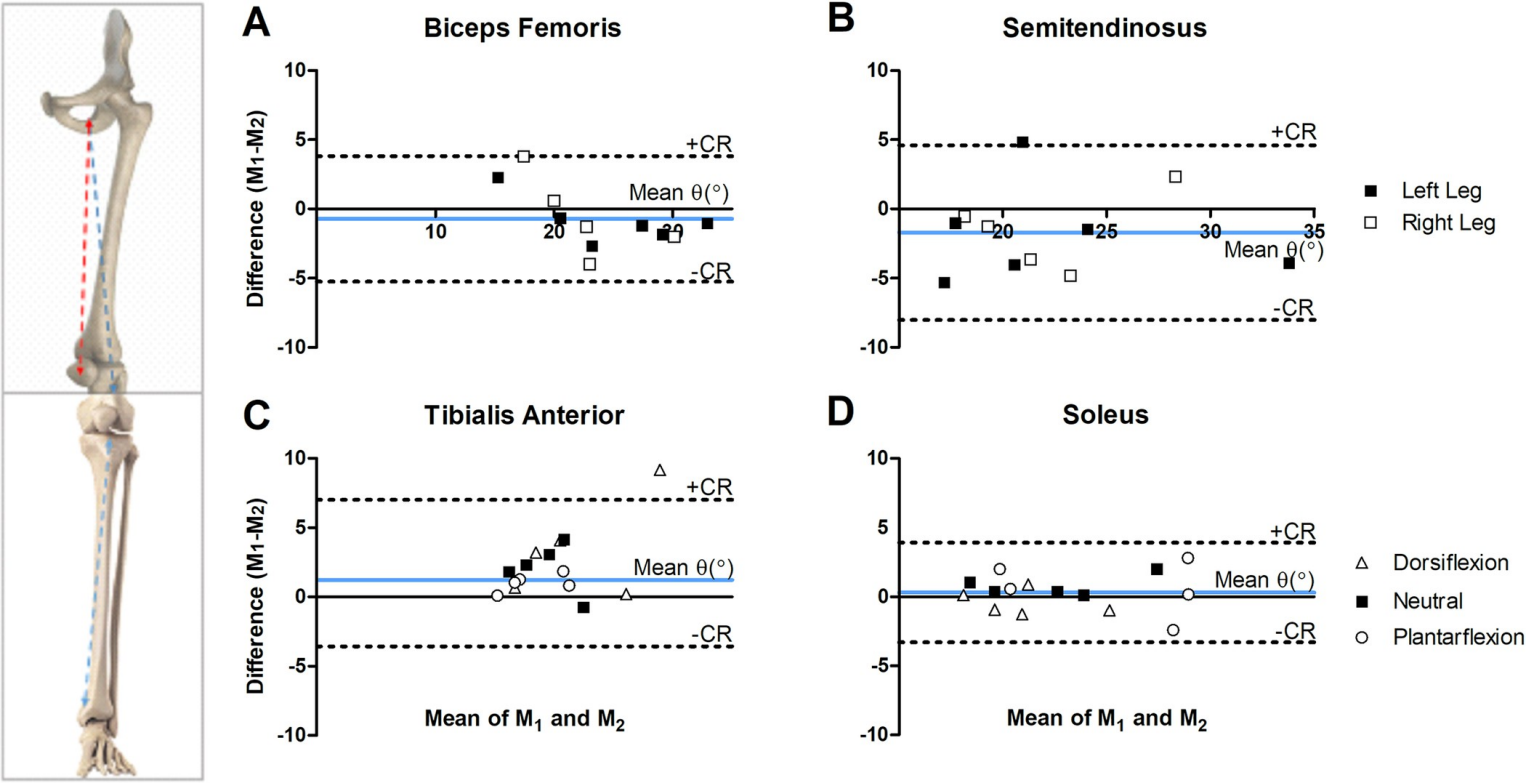
Bland-Altman analysis for the repeated measurements M1 and M_2_ of the whole volume average fiber angles θ_M1_ and θ_M2_ for **(A)** Biceps Fermoris, **(B)** Semitendinosus, **(C)** Tibialis Anterior, and **(D)** Soleus muscles. θ_M1_ and θ_M2_ are the repeated measurements. The light blue line indicates the bias and the dashed lines indicate the Bias±Coefficient of Repeatability (CR), *i*.*e*., ±1.96 x standard deviation (SD).

The Bland-Altman analysis of the whole-muscle mean fiber angle (θ) in the ST and BF of the right and left upper legs and the TA, SOL and anterior right, anterior left, posterior right and posterior left compartments of the SOL muscle in the lower leg are shown in Fig 2. The mean angle, the Bland-Altman analyses on the full muscle volumes and on the SOL anatomical compartments, the bias and SD, the CR, the mean CV%, MDD, ICC and SE are listed in Tables 1 and 2. For experiment 1 (upper leg muscles), the bias was -0.6° ± 2.9° in the right leg BF and -0.9° ± 1.7° in the left leg BF, with Coefficient of Repeatability(CR) ±5.7and ±7, respectively. For the ST, the bias was -1.6° ± 2.8° for the right leg and -1.8° ± 3.7° for the left leg, with CR ±5.5 and ±7.1 for right and left, respectively (Table 1). For the SOL muscle in experiment 2, the bias between the two measurements in neutral, dorsiflexion, and plantarflexion position was 0.8° ± 0.8°, 0.3° ±1.4°, and 0.6° ± 2.0° with CR ±1.8, ±1.5, and ±3.9 (Table 2). For the TA muscle, the bias was 2.1° ± 1.8°, 3.5° ± 3.6°, 1.0° ± 0.6° with CR between ±7.0, ±3.6, ±1.2, for neutral, dorsiflexion and plantarflexion positions, respectively (Table 2).

**Table 1:** Quantitative results of Bland-Altman analysis of repeated measurements of fiber angles θ (^o^).

	BFL Right	BFL Left	ST Right	ST Left
**Angle θ**	22.7±4.7	25.0±6.7	22.1±4.1	20.1±3.3
**Bias±SD**	-0.6 ± 2.9	-0.9 ± 1.7	-1.6 ± 2.8	-1.8 ± 3.7
**CR**	±5.7	±7	±5.5	±7.1
**Mean CV%**	7.7	5.1	7.8	11.4
**ICC**	0.9	0.9	0.9	0.8
**MDD**	7.5	11.5	8.2	11.5
**SE**	2.7	4.1	2.9	4.1

The mean and standard deviation (SD) of the bias, the coefficient of repeatability (CR) and the CV% are shown for the left and right Biceps Femoris Long Head (BFL).

**Table 2:** Quantitative results of Bland-Altman analysis of repeated measurements of fiber angles θ (^o^).

		TA	SOL	SOL ANT RIGHT	SOL ANT LEFT	SOL POST RIGHT	SOL POST LEFT
**Dorsiflexion**	**Angle θ**	20.0 ±4.4	21.4±1.7	15.3±2.0°	19.4±3.1°	24.6±2.1°	20.2±1.6°
	**Bias±SD**	1.5 ± 1.8	0.3 ± 1.4	0.5±1.4	2.6±3.1	0.6±3.1	1.2±1.9
	**CR**	±7.0	±1.8	±2.7	±6.1	3.9	±3.8
	**Mean CV%**	12.2	3.0	4.8	11.8	6.4	5.3
	**ICC**	0.7	0.8	0.9	0.5	0.03	0.4
	**MDD**	13.1	2.5	3.7	10.6	5.3	5.9
	**SE**	4.7	0.9	1.3	3.8	1.9	2.1
**Neutral**	**Angle θ**	17.4±2.2	21.7±1.6	15.9±1.9°	18.8±3.3°	24.7±1.7°	20.2±2.1°
	**Bias±SD**	1.2 ± 1.8	0.1 ± 1.0	0.3±1.3	0.8±1.9	0.8±1.3	0.7±0.3
	**CR**	±3.6	±1.5	±2.5	±3.8	±1.5	±0.6
	**Mean CV%**	10.4	2.6	4.7	6.3	3.0	2.4
	**ICC**	0.7	0.9	0.9	0.9	0.9	0.9
	**MDD**	7.4	2.9	3.2	5.3	2.9	2.0
	**SE**	2.7	1.0	1.2	1.9	1.0	0.7
**Plantarflexion**	**Angle θ**	16.6±2.1	23.8±2.9	17.57±3.4°	21.1±4.6°	26.0±5.2°	22.1±4.5°
	**Bias±SD**	1.0 ± 0.6	0.6 ± 1.5	1.0±3.6	2.4±3.7	-0.4±3.8	1.6±3.2
	**CR**	±1.2	±3.9	±7.1	±6.1	±1.8	±6.3
	**Mean CV%**	4.4	4.9	10.2	7.7	12.4	9.4
	**ICC**	0.9	0.8	0.7	0.8	0.7	0.8
	**MDD**	3.2	5.3	9.4	10.6	2.6	9.1
	**SE**	1.1	1.9	3.4	3.8	0.9	3.3

The mean and standard deviation (SD) of the bias, the coefficient of repeatability (CR), the CV% the ICC and the SE are shown for the left and right Semitendinosus (ST), the Tibialis anterior (TA), and the Soleus (SOL) muscle.

**Fig 3:**
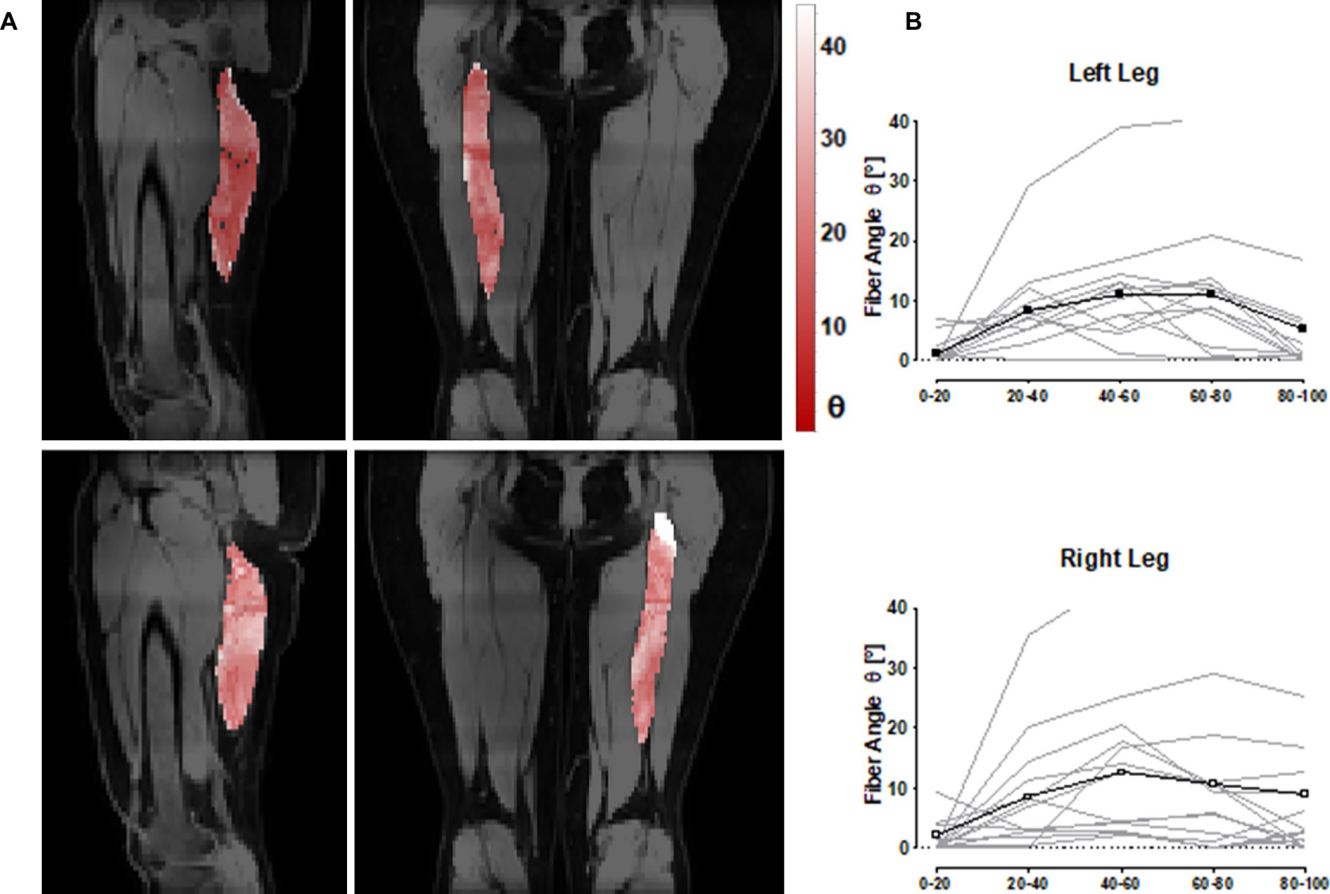
**(A)** 3D fiber angle color maps of the right (top) and left (bottom) semitendinosus (ST) muscles in sagittal and transversal views. **(B)** The mean fiber angle θ of the left and right ST muscles in five evenly distributed regions proximally to distally along the muscle length and normalized to the lowest value. The black curve represents the mean angle of the 10 measurements (5 subjects measured twice).

The ability of the method to detect differences in muscle fiber angles along the length of the muscle was assessed in the ST muscle. Fiber angle color maps of the ST muscles overlayed on the anatomical MR image together with the mean fiber angles in the 5 proximal to distal sub-sections are shown in Fig 3. The average measured fiber angle for the ST was 22.1° ± 4.1°. The muscle fiber angle distribution along the muscle length was highly subject dependent, with one subject having particularly high fiber angles in the medial portion (40–60%) of the ST muscle. Nevertheless, an average fiber angle distribution of the ST muscle was determined for all subjects, showing a parabolic curved angle distribution (Fig 3).

**Fig 4:**
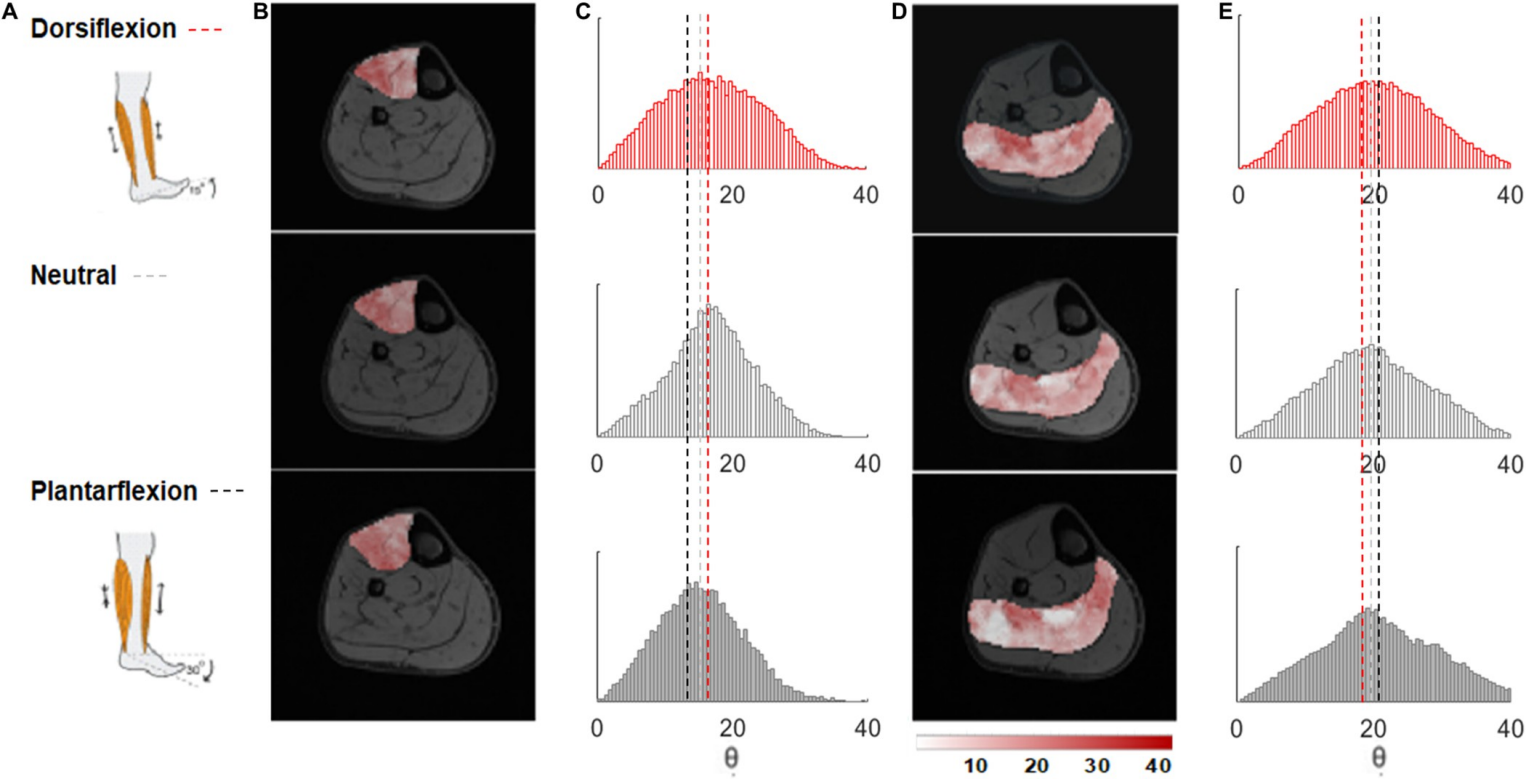
Fiber angle distributions of the TA and SOL muscles with different foot positions and fiber angle color maps for a representative subject. **(A)** Schematic drawing of dorsiflexion and plantarflexion position of the foot **(B)** Fiber angle color maps of the TA muscle overlayed on a mid-belly slice of the Dixon water image for the three foot.

**Fig 5:**
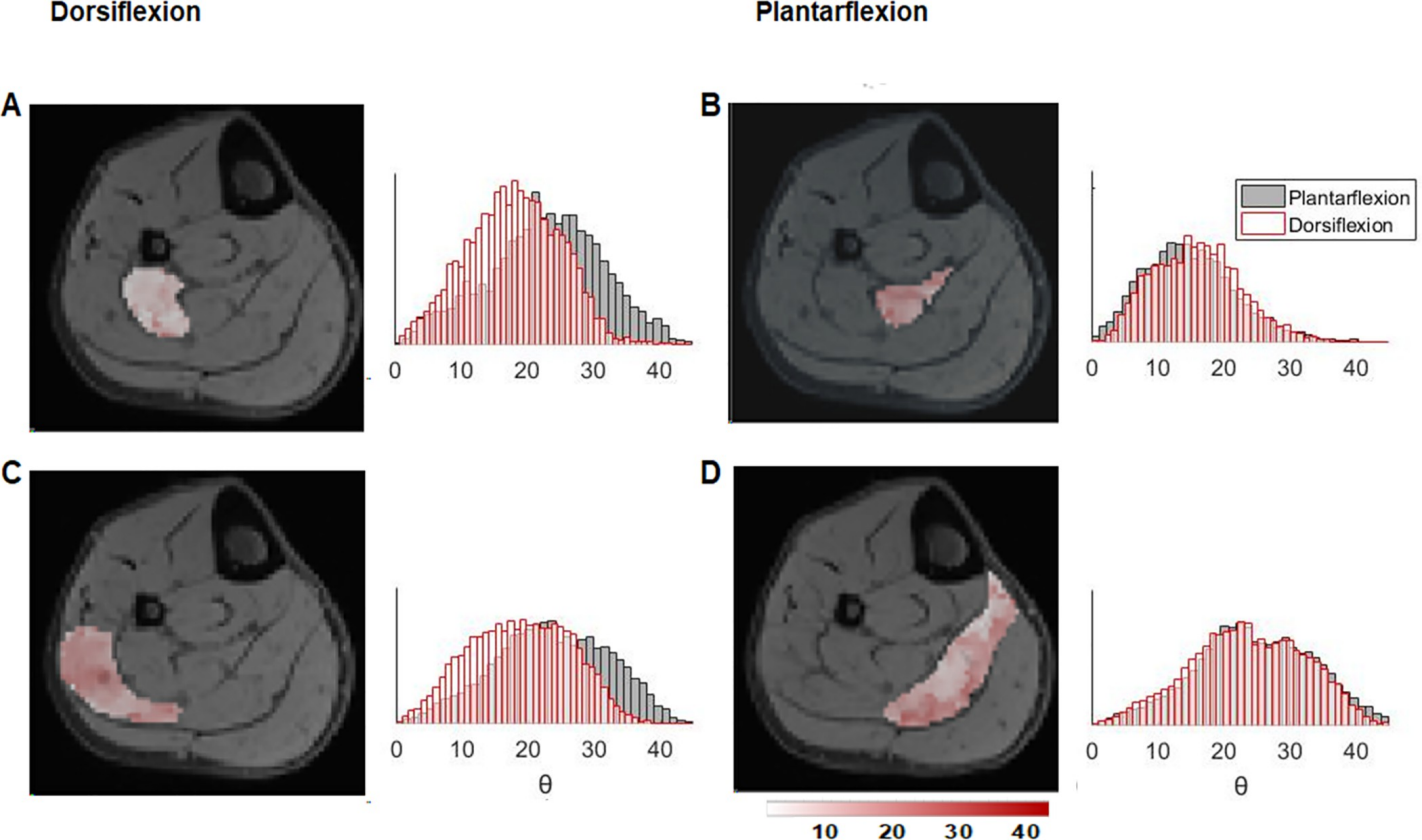
Fiber angle distributions of the SOL muscle with dorsiflexion and plantarflexion foot positions for a representative subject. The SOL muscle is segmented in 4 volumes with **(A)** the left anterior, **(B)** the right anterior, **(C)** the left proximal, and **(D)** the right proximal compartments. Color maps of the fiber angles are shown as overlays on a mid-muscle belly water Dixon image.

The evaluation of the method’s ability to detect small changes in fiber angles due to different ankle position using the fiber angle color maps and fiber angle distribution plots in the full muscle volumes of the TA and SOL muscles and in the four sub-compartments (two anterior (left & right) and two posterior (left & right)) of the SOL muscle is shown in Figs 4 and 5, respectively. Fig 4 shows, in line with the agonist and antagonist function of the TA and SOL muscles, that the fiber angle distribution of the full muscle volumes shift to higher θ for the TA muscle (20.0°) and lower θ for the SOL muscle (21.4°) in dorsiflexion position, whereas the distribution shifts to lower θ for the TA muscle (16.0°) and higher θ for the SOL muscle (23.8±2.9°) in plantarflexion position. Furthermore, regional differences in θ between ankle positions are visible in the fiber angle color maps for both the TA and the SOL muscles. For example, the anterior and posterior compartments of the SOL muscle become more distinctly visible during contraction (plantarflexion) as white and red patchy areas. Because of the complex multi-pennate architecture of the SOL muscle, we performed a detailed assessment of the changes in fiber angles with ankle position for the four sub-compartments. In nine out of ten measurements, the fiber angle distribution of the left compartments shifted to higher fiber angles with changing ankle position from dorsiflexion to plantarflexion, whereas the fiber angle distributions of the right compartments remained largely the same (Fig 5).

**Fig 6:**
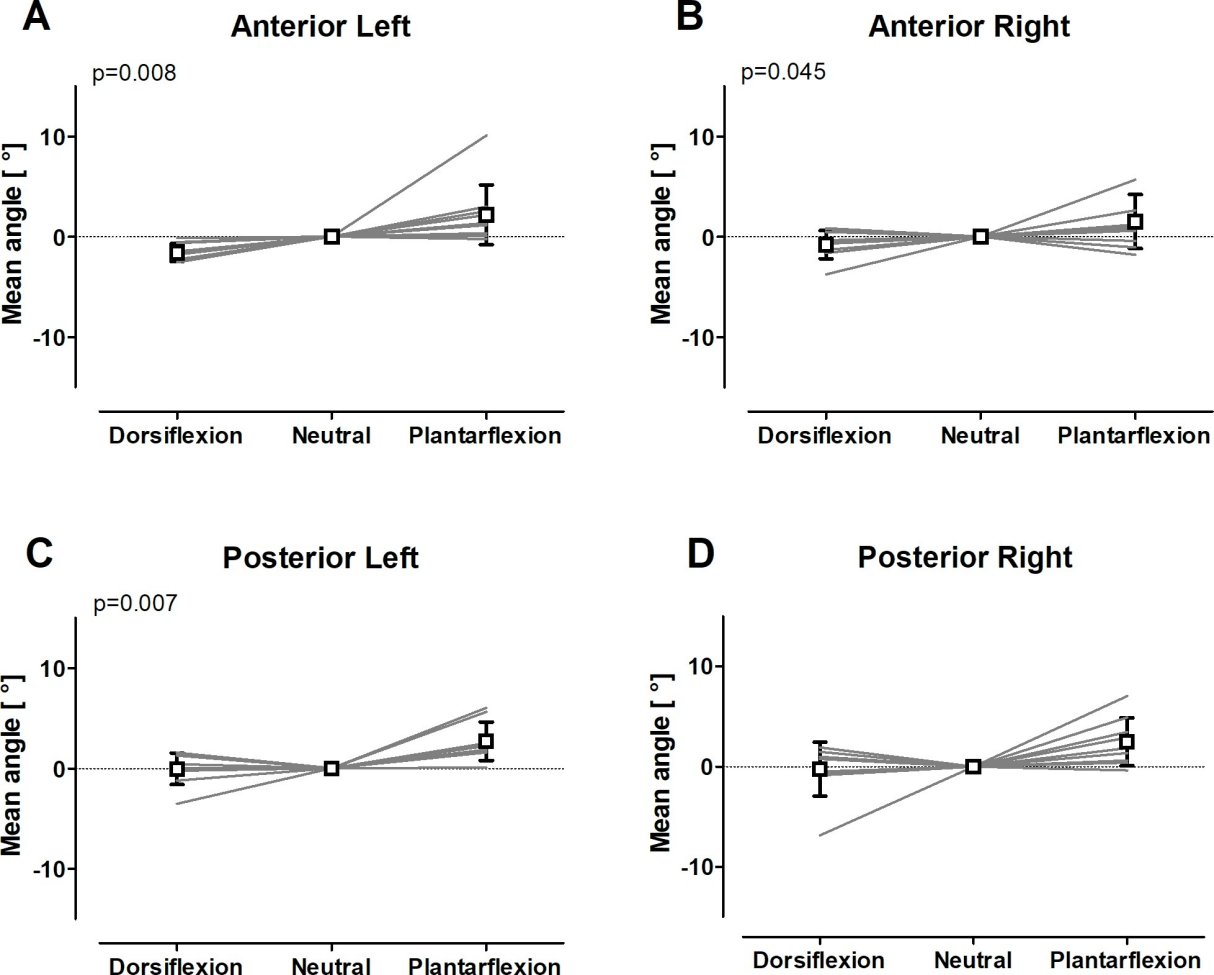
Mean fiber angles θ in the four sub-compartments of the SOL muscle as function of foot position. The fiber angles are shown for the **(A)** left anterior, **(B)** right anterior, **(C)** left posterior, and **(D)** right posterior compartments. Gray solid lines are the data for the individual subjects, whereas the open square symbols are group averaged and standard deviation.

The normalized changes of the mean fiber angles Δθ for dorsiflexion and plantarflexion with respect to the neutral position are summarized for the four compartments of the SOL muscle in Fig 6. The anterior and posterior left sub-compartments of the SOL muscle showed statistical difference between the three ankle positions, with the lowest angles in dorsiflexion position and highest angles in plantarflexion position. Table 3 shows the mean angle θ, the mean angle change Δθ and the p-value of the Friedman and Wilcoxon test in dorsiflexion and plantarflexion for 10 subjects in the four sub-compartments of the SOL muscle (anterior right, anterior left, posterior right, and posterior left).

**Table 3:** The mean measured fiber angle (Ɵ) in three ankle positions (dorsiflexion, neutral and plantarflexion), the mean change of angle (Δθ) in dorsiflexion and plantarflexion normalized in respect to the neutral position and the p-value of the Friedman Test and the post-hoc correction are reported among 10 subjects in the four compartments of the SOL muscle (anterior right, anterior left, posterior right and posterior left).

	Ankle Position	Anterior		Posterior	
		Right	Left	Right	Left
**Angle θ**	**Dorsiflexion**	15.3±2.0°	19.4±3.1°	24.6±2.1°	20.2±1.6°
	**Neutral**	15.9±1.9°	18.8±3.3°	24.7±1.7°	20.2±2.1°
	**Plantarflexion**	17.57±3.4°	21.1±4.6°	26.0±5.2°	22.1±4.5°
**Change Δθ**	**Dorsiflexion**	-0.8± 1.3°	0.6± 0.8°	-0.2± 2.5°	-0.0 ± 1.5°
	**Plantarflexion**	1.5± 2.6°	2.2± 2.8°	2.5± 2.2°	2.7±1.8°
**p-value**	**Friedman Test**	0.045	0.008	0.06	0.007
	**Wilcoxon test**		0.009		

The changes were statistically significant in the anterior and posterior left compartments (p = 0.008 and p = 0.007) and in the anterior right compartment (p = 0.045) of the SOL muscle. The more detailed post-hoc analysis showed significant differences Δθ = 2.2° ± 2.8° in fiber angles between the neutral and the plantarflexion positions in the anterior left sub-compartment of the SOL muscles (p = 0.009), in which the bias was 0.1°±1.0 and 0.6° ± 1.5°, whereas the CR was ±1.5 and ±3.9, suggesting that plantarflexion position contributed majorly to this main effect.

## Discussion

In this study we presented a DTI-based method to quantitatively assess fiber angles and changes therein in whole leg muscle volumes. We found that our method for quantification of fiber angle is feasible in the mean angle of the full volumes of both the lower and upper leg muscles. Furthermore, we showed that our method is sensitive to detect fiber angle differences along the length of the muscle and between different ankle positions.

Key to our method is that we defined the fiber angle in 3D and with respect to a reference line. This approach differs from the definition of 2D pennation angle which is the acute angle between the fascicles and the muscle aponeurosis or tendon [34]. The reference line is determined using two points, which are systematically defined on the basis of well visible anatomical landmarks, such as bones and tendons. The definition of the anatomical landmarks is set for each muscle, in the upper legs they were at the origo and insertion points, according to a simple definition of the line of action of the muscles [28, 35], whereas in the lower leg the reference line was based on the tibia bone. Consequently the reference line is rotationally invariant, which guarantees its high correspondence in a longitudinal follow up of the same subject, even in cases with severe remodeling.

The measurement of the pennation angle in the BFL and the ST muscles are in line with previous ultrasound studies that reported a pennation angle of 20.74° ± 2.53° as average of N = 8 locations in the BFL muscle [36] and 18.07° in the ST muscle for control subjects at rest [34].

Moreover, the ultrasound and cadaver study by Tosovic *et al*. [37] reported the same muscle angle curved distribution as the one we reported for the BFL and the ST muscles.

Our calculated fiber angles showed good repeatability in both the upper legs and the lower leg muscles because presented CV<10 and ICC **≥**0.7, with exception of the ST (left leg) and the TA muscle in dorsiflexion. The results are comparable to the repeatability reported in other DTI and US studies. In fact, in previous validation studies differences of 3.0° ± 7.3° between 2D ultrasound and human dissection in BFL were not considered significant, while changes of 6° were observed with knee and hip rotations of up to 90° [38]. Moreover, we believe that the goodness of the repeatability depends on the expected changes in the specific clinical application of the method. For instance, previous ultrasound studies in the BFL muscle after intervention reported a difference in the pennation angle in the BFL between re-injured and non-reinjured subjects of only 1.4° at the halfway point between the ischial tuberosity and the knee joint fold [39]. We think the ability to measure such change with and MRI-based method would be difficult. A possible improvement can be achieved by dividing the muscle length evenly and measuring the mean angle in the medial regions of the muscle length.

Of all analyzed muscles and positions, the muscle in the right upper leg and the TA in the dorsiflexion ankle position showed the largest CR. This might be due to the fact that there are shading artifacts in the left upper leg due to B1 inhomogeneity with the 3T wavelength. The lower repeatability of the TA muscle in dorsiflexion could be due to the fact that this ankle position was not well tolerated by all subjects resulting in some difficulty to control ankle position during and between measurements.

Froeling *et al*. [26] and Heemskerk *et al*. [21], in the assessment’s study of the reproducibility of DTI indices, identified as causes of lower repeatability multiple factors like the variations in anatomy (arms, legs) and muscles, repositioning, SNR and inclusion of non-muscle tissue, such as adipose tissue and blood vessels.

The good repeatability of our method is in line to the repeatability presented in studies on other fiber tractography based methods to quantify muscle pennation angle [16–18]. Previous studies have shown that the tractography repeatability is not optimal yet and that differences in the stopping criteria and the algorithm can influence the pennation angle calculation [39, 40, 41, 42].

Recent work by Bolsterlee *a*nd colleagues [16, 17] showed that anatomically constrained tractography can deal with some of the variations introduced by fiber tractography. To the best of our understanding, the exclusion of the fiber tracts was done on the basis of specific criteria such as the fiber length. Therefore, only a certain ratio of seeds, which was different for each muscle, led to successfully tracked fibers [17]. Furthermore, Bolsterlee *et al*. fitted fiber tracts to different polynomial orders, which resulted in minor mean absolute differences of the pennation angle between 0.5° and 1.9°. In two different studies by Bolsterlee and colleagues the angles in the individual compartments of the SOL muscle have been measured according to two different definitions.

In the first study [17], the angles were measured with respect to the long axis of the muscle, defined as a line connecting a proximal and a distal point on the anterior surface of the muscle between medial-anterior and lateral-anterior compartment. In the medial-anterior, lateral-anterior, medial-posterior and lateral-posterior the measured angles were 22°, 27°, 38°, 34° in plantarflexion with ankle angle 69°±12 and 17°, 18°, 24°, 24° in dorsiflexion with ankle angle of 108°±7. The pennation angles were larger in muscle plantarflexion than in dorsiflexion, like in our study. The difference with our measurements in the posterior compartments of the SOL muscle is due to the different degrees of flexion of the ankle, which varied between 19° and 56° instead of 45°.

In the second study by Bolsterlee and colleagues [16], the pennation angle was defined as 90° minus the mean angle between a vector parallel to the endpoint’s slope and the normal vectors of all triangles of the surface model within a radius of 1.5 mm around the endpoint. The pennation angle was the mean of the angles that the fascicle made with the deep and superficial aponeuroses. In this study the left foot was placed in neutral position with ankle angle 87°±3 with respect to the horizontal plane by mean of an MRI compatible foot plate. This study reported very similar results to the ones we measured: 19.0°, 20.0°, 24.6°, 21.2° in the medial posterior, medial anterior, lateral posterior and lateral anterior compartments.

This anatomically constrained method showed a good intra-class correlation coefficient (ICC) between 0.8 and 1 for pennation angle in the gastrocnemius muscle, but lower than in our study for the TA muscle with ICC of 0.6 and the anterior compartments of the SOL muscle in neutral position with ICC of 0.91, 0.14, 0.93, 0.60 in the medial-posterior, medial-anterior, lateral-posterior and lateral-anterior compartments. The lower repeatability in the TA muscle and anterior compartment of the SOL muscle compared to the other muscles is in line with our findings in the Bland Altman analysis. Even though the method with anatomical constraints proved repeatable and relatively insensitive to tracking parameters in the gastrocnemius muscle, it was not equally repeatable in the other muscles and several seed points (60%) and voxels of the full volume were excluded in order to obtain anatomically correct fibers.

In another study by Fouré and colleagues, a different fiber tractography based method was used [18, 40] and they defined 3D pennation angles between the principal axis of each muscle and the local muscle fiber direction. The principal axis was determined via a principal component analysis on the three-dimensional coordinates of each muscle segmentation mask. The local muscle fiber direction was taken from the first and last points of a fiber-tract in the muscle. Finally, the pennation angles were weighted over all fiber tracts within each voxel [39]. The calculated angle for the SOL muscle in eight young healthy subjects in 15°-20° plantarflexion was 43°± 3° and 29°± 6° for the superficial and deep compartments, respectively, which is around double of the value we found. These values are difficult to compare firstly because the SOL muscle was divided only in two compartments instead of four compartments, secondly because the change in angle between different positions was not assessed and finally, because in our experiment we used a reference line based on the tibia bone instead of the centroid of the muscle mask segmentation. Nevertheless, the difference between the superficial and deep compartment is comparable to the difference we found between the anterior right and the posterior right compartments of the SOL muscle in plantarflexion.

Furthermore, this method showed good reproducibility, reflected by low coefficient of variances between two measurements [18]. The mean CV% = 5.5 and 6.6 for the superficial (posterior) and the deep (anterior) SOL muscle and mean CV% = 8.4 for the TA.

The study investigated changes in pennation angle due to gender but not changes due to the effect of ankle position.

In our study, the evaluation of the effect of ankle positions on fiber angles showed significant differences in fiber angles between the neutral and the plantarflexion positions in the anterior left sub-compartment of the SOL muscles, suggesting that plantarflexion position contributed majorly to the statistical difference between the three ankle positions.

Our observations in the full volumes of both lower leg muscles are in agreement with biomechanical predictions. The fiber angle is expected to decrease during passive lengthening and to increase during passive shortening [43, 44]. Previous work by Sinha *et al*, using a method based on the first eigenvector with polar coordinates in respect to the magnet z-axis on six healthy subjects, reported changes in the fiber angles of the SOL muscle medialis up to 48° and 41° respectively in the anterior and in the posterior sub compartments between neutral (ankle angle 90°) and plantarflexion (ankle angle 120°) [45]. This change is much larger than we measured and is probably due to the different definition of pennation angle used and the area analyzed.

In contrast, changes in fiber angles between ankle positions in the sub-compartments of the SOL muscle we report here compare very well to those reported by Bolsterlee *et al*. who found changes in pennation angles in the range of approximately 5° to 14° for the different compartments of the SOL muscle [16]. Previous studies showed that architectural patterns during muscular shortening can be heterogeneous in the muscle and between subjects [43, 44] and that changes in muscle architecture due to ankle position may not follow a linear relationship [46, 47].

Our approach used the definition of pennation angles in the 3D volume of upper leg muscles with respect to a simple definition of the muscle’s line of action in the upper legs with extended legs and in respect to the tibia bone in the lower leg. This measurement can be used to calculate the muscle force [28, 35] as well as to monitor global or local changes of muscle fiber angles following, training or therapeutic intervention.

On the other hand, if a 2D definition of pennation angle would be desired instead in a specific region of the muscle the method here presented allows fast calculation of fiber angles with respect to new reference lines or planes conforming to other pennation angle definitions.

A limitation to our study is that we did not provide direct comparison with an independent technique like ultrasound, which can be considered the most established technique for the determination of fiber angles in skeletal muscle.

## Conclusions

In this study we have presented a method to assess fiber angles in skeletal muscle and changes therein. Our study indicated that fiber angle quantification in full 3D volumes and in specific muscle compartments of individual muscles is feasible with this method. Additionally, this method is sensitive enough to detect fiber architecture changes in lower leg muscles due to different foot positions. These results warrant the application of our method to longitudinally monitor changes in fiber angles following training, intervention and to assess response to treatment.

## Supporting information

S1 File BFL right. Mean of repeated measurements of fiber angles θ (^o^) in the BFL muscle of the right leg.
(XLSX)

S2 File BFL left. Mean of repeated measurements of fiber angles θ (^o^) in the BFL muscle of the left leg.
(XLSX)

S3 File ST right. Mean of repeated measurements of fiber angles θ (^o^) in the ST muscle of the right leg.
(XLSX)

S4 File ST left. Mean of repeated measurements of fiber angles θ (^o^) in the ST muscle of the left leg.
(XLSX)

S5 File SOL. Mean of repeated measurements of fiber angles θ (^o^) in the SOL muscle.
(XLSX)

S6 File TA. Mean of repeated measurements of fiber angles θ (^o^) in the TA muscle.
(XLSX)

S7 File SOL ANT RIGHT. Mean of repeated measurements of fiber angles θ (^o^) in the SOL muscle anterior right compartment.
(XLSX)

S8 File SOL ANT LEFT. Mean of repeated measurements of fiber angles θ (^o^) in the SOL muscle anterior left compartment.
(XLSX)

S9 File SOL POST RIGHT. Mean of repeated measurements of fiber angles θ (^o^) in the SOL muscle posterior right compartment.
(XLSX)

S10 File SOL POST LEFT. Mean of repeated measurements of fiber angles θ (^o^) in the SOL muscle posterior left compartment.
(XLSX)

## Abbreviations list

BFL: Biceps Femoris Long Head Muscle
ST: Semitendinosus Muscle
SM: Semimembranosus Muscle
TA: Tibialis Anterior Muscle
SOL: Soleus Muscle
MD: Mean Diffusivity
RD: Radial Diffusivity
DTI: Diffusion Tensor Imaging
SNR: Signal-to-Noise Ratio
qT2: quantitative T2
λ1: Eigenvalue 1
λ2: Eigenvalue 2
λ3: Eigenvalue 3
FA: Fractional Anisotropy
FACM: Fascicle-angle color maps
Ɛ1: Eigenvector 1
