# Correction: A diffusion tensor-based method facilitating volumetric assessment of fiber orientations in skeletal muscle

**DOI:** 10.1371/journal.pone.0314063

**Published:** 2024-11-13

**Authors:** Laura Secondulfo, Melissa T. Hooijmans, Jozef J. M. Suskens, Valentina Mazzoli, Mario Maas, Johannes L. Tol, Aart J. Nederveen, Gustav J. Strijkers

The third author’s name is spelled incorrectly. The correct name is: Jozef J M Suskens. The correct citation is: Secondulfo L, Hooijmans MT, Suskens JJM, Mazzoli V, Maas M, Tol JL, et al. (2022) A diffusion tensor-based method facilitating volumetric assessment of fiber orientations in skeletal muscle. PLoS ONE 17(1): e0261777. https://doi.org/10.1371/journal.pone.0261777.
